# Characterization of Responsive Hydrogel Nanoparticles upon Polyelectrolyte Complexation

**DOI:** 10.3390/polym9020066

**Published:** 2017-02-16

**Authors:** Su-Kyoung Lee, Gyuri Hwang, Jihyun Woo, Joseph Park, Jongseong Kim

**Affiliations:** 1 Yonsei-IBS Institute, Yonsei University, Seoul 03722, Korea; switfrik@gmail.com; 2 STEM Research Institute, Fairfax, VA 22031, USA; paulschung08@gmail.com (G.H.); joo1123kr@gmail.com (J.W.); hydrom7@daum.net (J.P.)

**Keywords:** hydrogel, nanogel, polyelectrolyte, aggregation, deswelling, swelling

## Abstract

Characterization of responsive hydrogels and their interaction with other molecules have significantly expanded our understanding of the functional materials. We here report on the response of poly(*N*-isopropylacrylamide-*co*-acrylic acid) (pNIPAm-*co*-AAc) nanogels to the addition of the poly(allylamine hydrochloride) (PAH) in aqueous dispersions. We find that the hydrodynamic radius and stability of nanogels are dependent on the PAH/nanogel stoichiometry. If the nanogel solution is titrated with very small aliquots of PAH, the nanogels decrease in radius until the equivalence point, followed by aggregation at suprastoichiometric PAH additions. Conversely, when titrated with large aliquots, the nanogel charge switches rapidly from anionic to cationic, and no aggregation is observed. This behavior correlates well with electrophoretic mobility measurements, which shows the nanogel charge transitioning from negative to positive upon PAH addition. The volume phase transition temperature (VPTT) of the nanogels is also measured to discover the effect of polyelectrolyte complexation on the deswelling thermodynamics. These data show that charge neutralization upon PAH addition decreases the VPTT of the nanogel at pH 6.5. However, if an excess amount of PAH is added to the nanogel solution, the VPTT shifts back to higher temperatures due to the formation of a net positive charge in the nanogel network.

## 1. Introduction

Stimuli-responsive hydrogels have been investigated on various purposes due to the capability of hydrogels in response to external stimuli by physicochemical changes in the materials [1,2,3,4,5,6,7,8,9]. This responsivity is manifested in the swelling/deswelling of the polymer network as a function of temperature or pH [10,11,12]. In the swollen state, the polymer-solvent interactions are favorable, where in the specific case of poly(*N*-isopropylacrylamide) (pNIPAm), these interactions are favorable below the VPTT of 31 °C, which is analogous to the coil-to-globule transitions seen in linear polymer systems as the lower critical solution temperature (LCST) is exceeded [13]. AAc copolymers are additionally pH-responsive, where at pH values higher than p*K*_a_ (4.25) [14] of AAc, the network tends to swell further due to Coulombic repulsion between the deprotonated acid groups, also resulting in an increase in the VPTT [15]. The favorable polymer–water interactions in pNIPAm can be disrupted by increasing the temperature of the system above the VPTT. Under these conditions, it is entropically favorable for the network to expel water and hydrophobically aggregate, resulting in a large-magnitude decrease in the network volume. The swelling/deswelling due to changes in pH is less abrupt, where the responsivity is due to Coulombic repulsion and an increase in the Donnan potential inside the network as the AAc groups become charged. It has also been reported that specifically engineered hydrogels with additional functionalities are responsive to stimuli such as pH, ionic strength, photon flux, and biomolecular binding events [16,17,18]. These responsive hydrogels have been studied extensively for numerous applications, such as controlled drug release [19,20,21,22], tissue regeneration [5], surface patterning [23], microfluidic flow control [4], tunable optics [7,16,17,24], and molecular switches [25].

Polyelectrolyte complexation is an intriguing means useful for many applications by taking advantage of simple charge–charge interactions between polyelectrolytes, which includes not only the formation of physically cross-linked hydrogel particles [26,27] in drug delivery [28,29,30] but also fabrication of micro-capsules by building up polymeric multilayers on colloidal nanoparticles [31,32], followed by nanoparticle dissolution [33,34,35]. This phenomenon can also be exploited for the fabrication of thin films by the layer-by-layer (LbL) deposition approach. In addition, polyelectrolyte complex formation, known as complex coacervation, has been investigated to improve the applications [36], providing many interesting properties including low interfacial energy [37,38] and viscoelastic response [39]. Polyelectrolyte complex systems are extensively utilized to improve biological applications in drug delivery [40,41], tissue engineering [42], and biological hybrid thin films [43,44]. Polyelectrolyte complexation in application of polymeric thin films composed of pNIPAm and AAc allows for determining the interaction/effects of various polyelectrolytes on the behavior (swelling and VPTT) of the thin film [45,46]. Polyelectrolytes can also be exploited for gene delivery where electrostatic interactions between the polyelectrolyte and DNA are used to form nanoparticles which can transfect cells [47,48].

In this study, we explore the effect of added poly(allylamine hydrochloride) (PAH; p*K*a = 9.67) [49] on the colloidal stability and thermoresponsivity of pNIPAm-*co*-AAc nanogels. The pNIPAm-*co*-AAc nanogels behave as polyanions and reservoirs for polycation adsorption, allowing for their complexation in aqueous dispersion.

## 2. Materials and Methods

### 2.1. Materials

All reagents were purchased from Sigma-Aldrich unless otherwise specified. The monomer *N*-isopropylacrylamide (NIPAm) was re-crystallized from hexanes (J.T. Baker^®^, Center Valley, PA, USA) and dried under vacuum prior to use. Acrylic acid (AAc) was distilled under reduced pressure. The cross-linker *N*,*N*′-methylene(bisacrylamide) (BIS), ammonium persulfate (APS), and sodium dodecyl sulfate (SDS) were used as received. Poly(allylamine hydrochloride) (PAH), *M*_w_ 70,000, was used as received. All water used throughout this investigation was house distilled, deionized to a resistance of at least 18 MΩ (Barnstead Thermolyne E-Pure system, Thermo Fischer Scientific, Waltham, MA, USA), and then filtered through a 0.2 μm filter for particulate removal.

### 2.2. Nanogel Synthesis

All nanogels used throughout this study were synthesized via aqueous free-radical precipitation polymerization, as previously described [50,51,52]. The syntheses were carried out by fixing the total monomer concentration constant at 70 mM and the AAc mole percent constant at 10% while varying the BIS mole percent between 2% and 10% and adjusting the NIPAm concentration accordingly. Polymerization was performed in a three-neck, 250 mL round-bottom flask containing a magnetic stir bar. To this reactor, 100 mL of a filtered (0.2 μm filter, Pall Gelman Metricel, Port Washington, NY, USA), aqueous solution of NIPAm (697 or 622 mg), BIS (22 or 108 mg), and the surfactant SDS (28.8 mg, 1 mM final concentration) was added. This solution was heated to ~70 °C while degassing with N_2_ and stirring vigorously for ~1 h. After 1 h, AAc was added to the flask in order to bring the total final monomer concentration up to 70 mM. Once the AAc was added, polymerization was immediately initiated by injection of 1.0 mL of an APS solution (0.3 mmol). The solution turned turbid, indicating successful initiation. This solution was allowed to heat and stir for an additional 5 h while under a constant flow of N_2_ gas. Following synthesis, the particles were purified by dialysis against water for ~2 weeks with the water being changed twice per day, using 10,000 *M_W_* cut-off dialysis tubing (VWR).

### 2.3. Nanogel Titration with PAH

Titration of the nanogels used in this study was accomplished by adding aliquots of 0.0526 monoM (moles/L monomer) PAH solution (pH 4.2 with 10 mM NaCl) to a dilute solution of nanogels (10 μL of purified nanogels diluted to 3.5 mL), which is 2.0 × 10^−5^ M AAc, at pH 6.5 (adjusted by HCl and NaOH with 10 mM NaCl) contained in a plastic cuvette. Each nanogel solution was made immediately before each measurement. The radius and thermoresponsive behavior of the nanogels following titration was measured using Dynamic Light Scattering instrument (DLS, Protein Solutions Inc., Charlottesville, VA, USA).

### 2.4. Dynamic Light Scattering (DSL) Analysis

The use of DLS as a tool for determining mean particle size and particle size distributions has been described previously [53] and used for these nanogel systems in the past [50,52]. Briefly, the nanogel solution contained in a cuvette was inserted into the cuvette holder, which was equipped with a Peltier device for sample temperature control. Laser light (783.9 nm) was introduced to the sample via a single-mode optical fiber. Scattered light was collected by a fiber coupled avalanche photodiode detector at 90°. The scattered light can then be correlated to the translational diffusion coefficient of the nanogels, through the autocorrelation function, from which the hydrodynamic radius is calculated from the Stokes–Einstein equation. For equilibrium nanogel radius measurements, each sample was allowed to equilibrate at 25 °C for 10 min. The reported radius value was an average of 15 individual radius measurements using a 5 s integration time for each measurement. For VPTT measurements, the nanogels were first titrated with a given amount of PAH. The solution was then heated and the nanogel size determined every 2 °C by letting the sample equilibrate at each temperature for 10 min. At each temperature, 5 consecutive runs were performed where each run was composed of 15 individual radius measurements using a 10 s integration time for each measurement.

### 2.5. Electrophoretic Mobility Measurements

Electrophoretic mobility was measured to follow nanogel charge upon PAH addition using a ZetaPlus instrument (Brookhaven Instruments Corporation, Holtsville, NY, USA). The samples were prepared in the same way they were prepared for DLS analysis and each reported electrophoretic mobility value is the mean value of 10 runs. The software provided by the instrument manufacturer was used to calculate the electrophoretic mobility.

## 3. Results and Discussion

**Figure 1:**
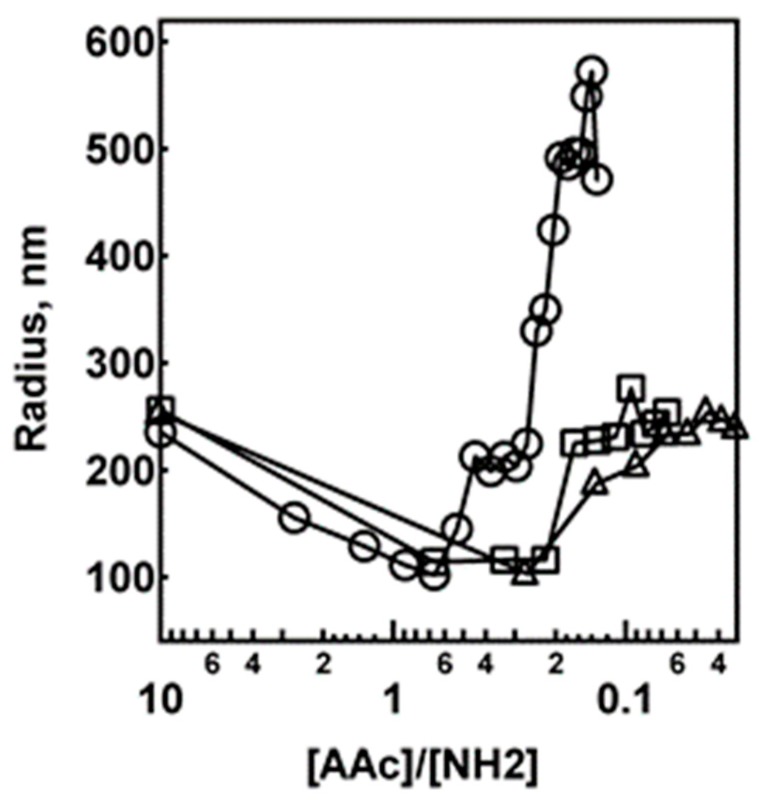
Nanogel titration plots for the titration of 2% BIS, 10% AAc nanogels upon the addition of 0.5 (**◯**), 2.0 (**□**), and 5.0 (**△**) L PAH aliquots. The hydrodynamic radius of nanogels measured by DLS.

The average nanogel hydrodynamic radius (*R_h_*) was determined by DLS as a function of PAH addition. As shown in Figure 1, for titration of 10% AAc/2% BIS nanogels, the radius decreases from ~250 to ~100 nm upon the addition of PAH, regardless of the PAH aliquot volume. In the case of 2.0 and 5.0 μL PAH aliquot additions, the nanogel size then increases upon subsequent PAH addition to a radius of ~250 nm. This value is important to note because it is approximately the same as the particle radius at this pH in the absence of PAH. This swelling behavior is most likely due to the nanogel network structure becoming internally cross-linked by AAc/NH_2_ interactions, followed by PAH saturation. The saturation of the particle with PAH should in turn produce an excess positive charge in the nanogel network, thereby resulting in Coulombic swelling. Another interesting feature in Figure 1 is the behavior seen upon adding 0.5 μL PAH aliquots to the system. As the [AAc]/[NH_2_] ratio approaches 1, the nanogel radius decreases, as seen in the case of the 2.0 and 5.0 μL PAH aliquot additions. However, in the case of 0.5 μL PAH aliquot addition, the nanogels tend to significantly increase in radius, indicating system aggregation rather than Coulombic swelling. Further evidence of aggregation comes from the dramatic increase in population breadth, as shown in the particle size histograms obtained from DLS (Figure S1). It is also interesting to note that there is no time dependency to the radius determination below the critical point, i.e., excess negative charge on the pNIPAm-*co*-AAc nanogel (Figures S2 and S3). As the point of neutrality is approached, the nanogels behave differently. The nanogel titrated with 0.5 μL PAH aliquot shows aggregation in a time-dependent manner (Figure S2), whereas in the case of 5.0 μL PAH aliquot the nanogel remains in no time-dependent kinetics and aggregation (Figure S3). This is an indication that the nanogels are becoming instantly charge-reversed upon the addition of saturating amounts of PAH.

One way to explain this behavior is by considering the fact that, as the nanogels reach the neutralization point, the electrical repulsion between the nanogel particles is decreased and van der Waals attraction may dominate the nanogel dispersion in aqueous system. Thus, the energy barrier that prevents particle collisions may be decreased resulting in aggregation of the nanogels on the time scale of the experiment [54,55,56]. However, this mechanism is not the most likely source of aggregation, since water-swollen pNIPAm nanogels dispersed in water should have a low effective Hamaker constant and do not tend to aggregate at temperatures below the VPTT. Another possible explanation of this behavior relates to the homogeneity (or lack thereof) of absorption of PAH on the nanogels. It is most likely the case that the absorption of PAH is inhomogeneous as one slowly approaches the stoichiometric equivalence point, resulting in nanogels that have patchy-type charge distribution. Thus, when the nanogels collide they should have a higher tendency to aggregate at slightly suprastoichiometric AAc/NH_2_ values [55,57].

**Figure 2:**
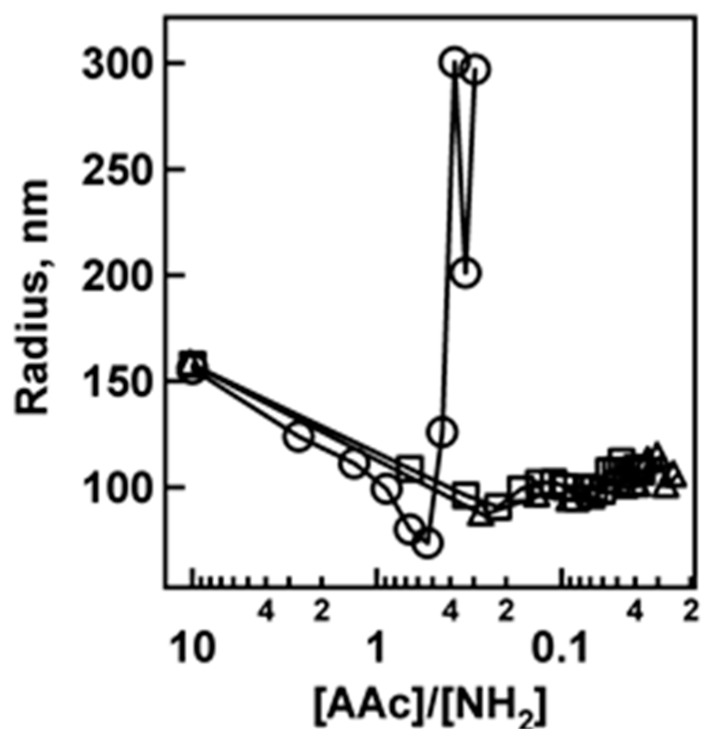
Nanogel titration plots for the titration of 10% BIS, 10% AAc nanogels upon the addition of 0.5 (**◯**), 2.0 (**□**), and 5.0 (**△**) μL PAH aliquots. The hydrodynamic radius of nanogels measured by DLS.

To determine if nanogel cross-link density has an effect on the interactions between PAH and AAc, titration experiments were conducted exactly as outlined above using nanogels containing 10% BIS and 10% AAc. As shown in Figure 2, the same qualitative trends are obtained as were seen for the 2% BIS titrations. For the 2.0 and 5.0 μL PAH aliquot additions, the nanogels are colloidally stable at all stoichiometries, but in the case of the 0.5 μL PAH aliquot addition, the nanogels again aggregate. One important difference that must be pointed out between the 10% and 2% BIS nanogel titrations is the magnitude of the deswelling upon PAH addition. In the 2% BIS case, the magnitude of collapse is much greater than that for the 10% BIS nanogels upon PAH addition. This is expected, as the 10% BIS nanogels have a greater network density than the 2% BIS nanogels, thus allowing for a smaller change in equilibrium swelling degree upon the addition of PAH.

**Figure 3:**
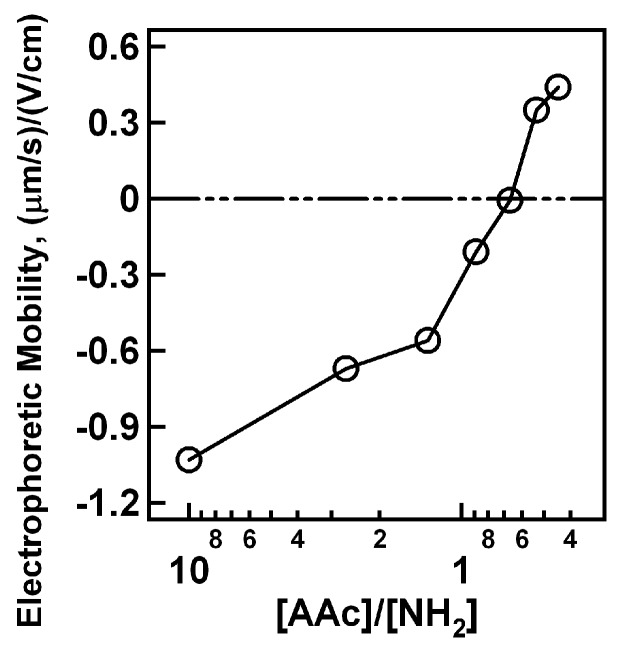
Electrophoretic mobility values as a function of PAH addition for 2% BIS and 10% AAc nanogels upon 0.5 μL PAH aliquot additions. Note the pH of solutions adjusted at 6.5 with 10 mM NaCl.

**Figure 4:**
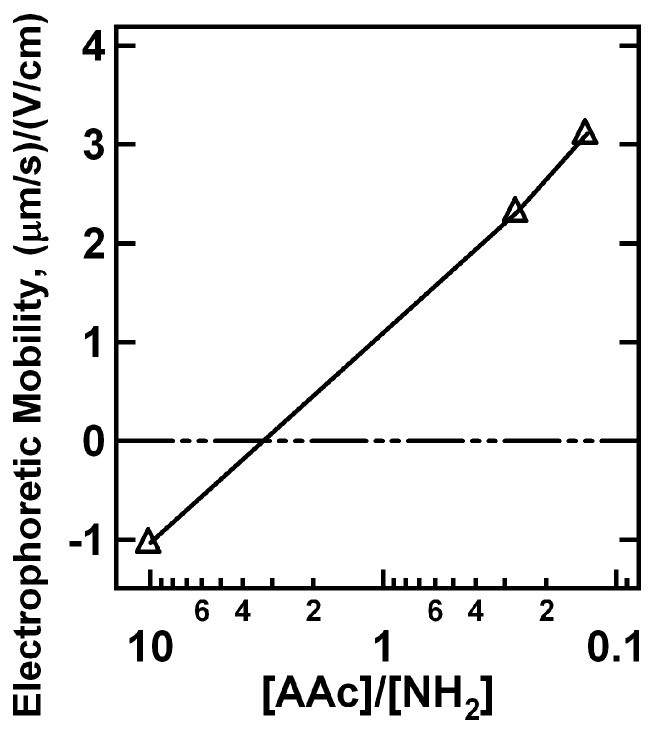
Electrophoretic mobility values as a function of PAH addition for 2% BIS and 10% AAc nanogels upon 5.0 μL PAH aliquot additions. Note the pH of solutions adjusted at 6.5 with 10 mM NaCl.

Electrophoretic mobility measurements were made for the pNIPAm-*co*-AAc nanogels containing 2% BIS upon the addition of 0.5 and 5.0 μL PAH aliquots to verify the above claims of nanogel neutralization and charge reversal. To interpret the data it is important to keep in mind that uncharged (no AAc) pNIPAm nanogels have approximately zero electrophoretic mobility under the conditions that these experiments were conducted (Figure S4). The data in Figure 3 show that the nanogels initially display a negative electrophoretic mobility due to the deprotonated AAc groups on the pNIPAm-*co*-AAc nanogels. As 0.5 μL PAH aliquots are titrated into the nanogel solution, the electrophoretic mobility slowly approaches zero. At ~1:1 AAc/NH_2_, the average electrophoretic mobility is approximately zero, as expected for a 1:1 acid/base stoichiometry. As more PAH is added to the system, the electrophoretic mobility then reverses to positive values due to the excess charge present in the nanogel. If, on the other hand, the electrophoretic mobility is followed as a function of 5.0 μL PAH aliquot additions, the behavior is very different. This result is shown in Figure 4, where following the first 5.0 μL PAH aliquot the average electrophoretic mobility reverses from a negative to a positive value. In light of these results, the data shown in Figure 1 and Figure 2 are understandable in terms of effective surface charge arguments. If excess amounts of PAH are added to the system (2.0 and 5.0 μL case) the nanogel rapidly reverses charge from negative to positive while never reaching an equilibrated point of instability (neutrality). In contrast, 0.5 μL PAH aliquot additions cause the critical aggregation (neutralization) point to be reached slowly, whereupon the nanogels are able to aggregate due to the inhomogeneous charge distribution present on the nanogels.

**Figure 5:**
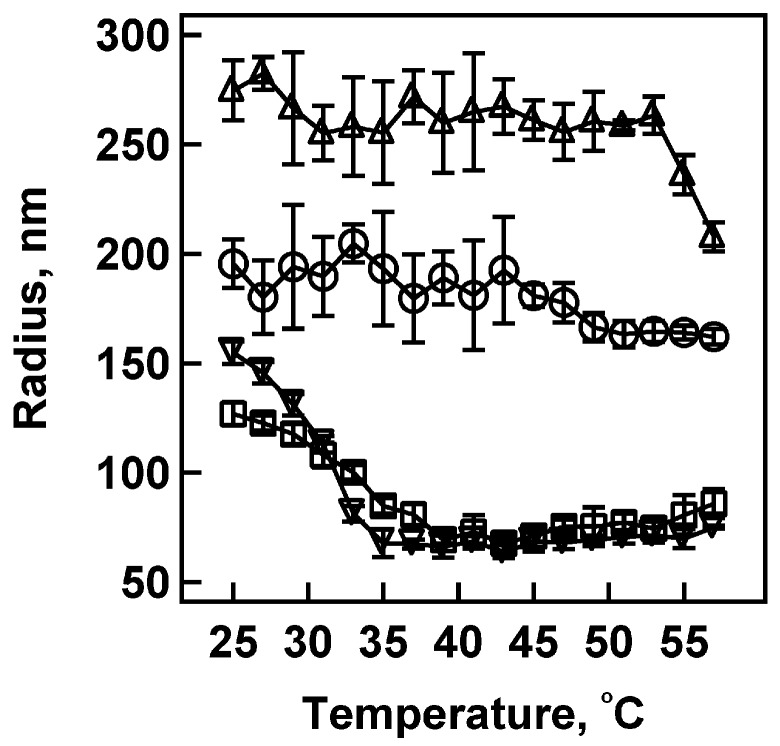
Volume phase transition curves for 2% BIS, 10% AAc nanogels at pH 6.5 (**△**), pH 3.0 (**▽**), pH 6.5 with [AAc]/[NH_2_] = 0.9 (**□**), and pH 6.5 with [AAc]/[NH_2_] = 0.26 (**◯**). The VPTT for nanogels at pH 6.5 with [AAc]/[NH_2_]= 0.9 is very close to the VPTT observed for pH 3.0.

To investigate the influence of PAH on the thermal deswelling of pNIPAm-AAc nanogels, we determined the particle size as a function of temperature at various nanogel/PAH stoichiometries (Figure 5). This figure shows a series of volume phase transition curves obtained by DLS for pNIPAm-*co*-AAc nanogels at pH 3.0, where the particles are essentially electroneutral, and at pH 6.5, where the particles are anionic due to AAc deprotonation. As shown in Figure 5, the pNIPAm-*co*-AAc nanogels at pH 3.0 display a VPTT of 30 °C, while at pH 6.5 shifting the value to ~54 °C [12,52,58]. However, when pNIPAm-*co*-AAc nanogels at pH 6.5 are titrated with PAH such that AAc/NH_2_ is ~1:1, the nanogel VPTT reverts to 33 °C. This behavior suggests that the nanogel structure is becoming fully neutralized due to the AAc–NH_2_ interactions, where the NH_2_ groups are capable of neutralizing the deprotonated AAc sites inside the nanogel network. It is also interesting to note that the nanogel radius following PAH addition is somewhat smaller that the radius of the native nanogel at pH 3.0 (125 nm vs. 150 nm), suggesting that, in addition to charge neutrality, an effective increase in cross-link density arises from polyelectrolyte complexation. Figure 5 also shows the effect of the addition of excess PAH to the pNIPAm-*co*-AAc nanogels, where no VPTT is observed over this temperature range. This difference is most likely due to the excess PAH contributing to a net positive charge inside the nanogel structure, resulting in Coulombic repulsion inside the nanogel network, which then shifts the VPTT to a higher temperature. Our electrophoretic mobility results are in part suggestive of this, as it is clear that suprastoichiometric concentrations of PAH induce charge reversal. The DLS results additionally suggest that this charge reversal is not solely localized on the nanogel surface, as simple surface charging would not be expected to inhibit nanogel deswelling on the whole. For example, multiple papers on pNIPAm core/pNIPAm-*co*-AAc shell nanogels have shown that highly charged nanogel shells do not completely inhibit core deswelling. This observation indicates that charge reversal of the nanogels is accomplished with excess PAH addition.

## 4. Conclusions

We have illustrated that loosely cross-linked pNIPAm-*co*-AAc nanogels in their deprotonated form bind with the linear polyelectrolyte PAH via ion pairing interactions. Light scattering and electrophoretic mobility measurements suggest that the hydrodynamic radius of pNIPAm-*co*-AAc nanogels is predominantly influenced not only by the cross-linking density of the hydrogels but also by the net charge of the gel network. This causes dramatic deswelling of the gels due to both charge compensation and cross-linking effects. It has also been demonstrated that the colloidal stability of the nanogels is impacted by the rate at which PAH is added to the dispersion. By approaching the point of stoichiometric equivalence slowly, a point of zwitterionic charge neutrality is obtained, resulting in particle aggregation. Conversely, large increases in PAH concentration overshoot this instability point and allow for charge reversal on the nanogel particle. Observing VPTT shifts as a function of PAH addition corroborates this behavior. When appropriate PAH is present to neutralize the charges inside the nanogels, the VPTT shifts to temperatures observed for neutralized nanogels at pH 3.0. If an excess amount of PAH is added into the nanogel solution, the VPTT shifts back to high temperatures illustrating the charge reversal phenomenon.

## Supplementary Materials

The following are available online at www.mdpi.com/2073-4360/9/2/66/s1: Figure S1: Histograms for the 0.5 and 5.0 μL PAH aliquot addition cases for 2% BIS cross-linked nanogels; Figure S2: Time dependent nanogel titration plot for the 2% BIS, 10% AAc nanogels upon the addition of 0.5 μL PAH aliquots at 0 (**◯**), 210 (**□**), 420 (**△**), and 630 (**▽**) s; Figure S3: Time dependent nanogel titration plot for the 2% BIS, 10% AAc nanogels upon the addition of 5 μL PAH aliquots at 0 (**◯**), 210 (**□**), 420 (**△**), and 630 (**▽**) s; Figure S4: Electrophoretic mobility values as a function of PAH addition for 2% BIS, 98% NIPAm nanogels.
