# Supplementary material for: Characterization of Responsive Hydrogel Nanoparticles upon Polyelectrolyte Complexation
Source: Polymers (Basel). 2017 Feb 16;9(2):66. doi: 10.3390/polym9020066 (PMC6431993; doi:10.3390/polym9020066)
Supplement: Supplementary file 1 [file polymers-09-00066-s001.pdf]

Supplementary Materials: Characterization of Responsive Hydrogel Nanoparticles upon Polyelectrolyte Complexation

Su-Kyoung Lee, Gyuri Hwang, Jihyun Woo, Joseph Park and Jongseong Kim

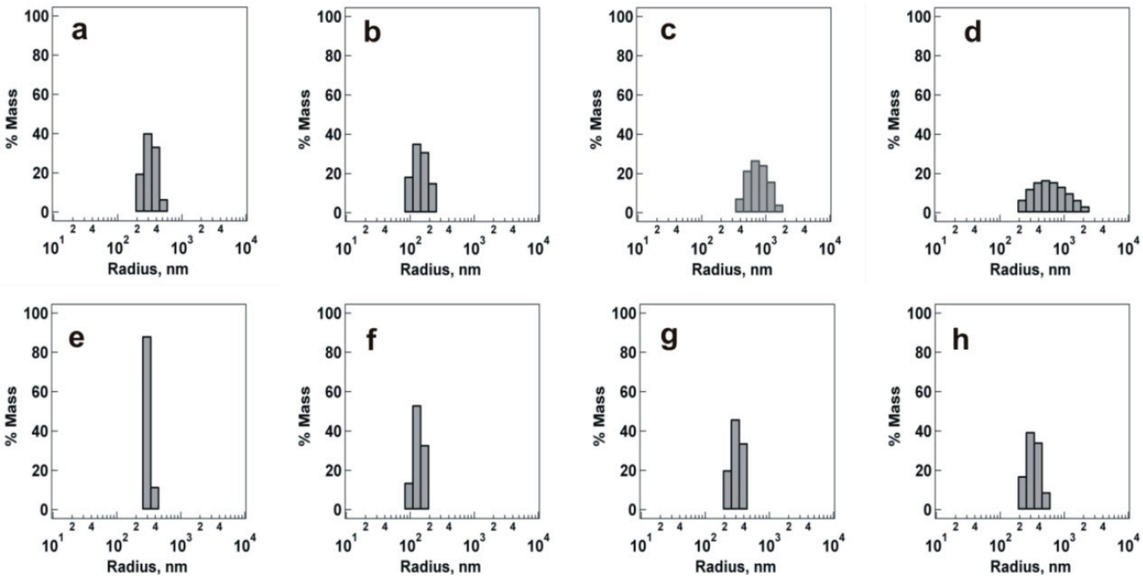

Figure S1. Histograms for the 0.5 and 5.0 L PAH aliquot addition cases for 2% BIS cross-linked microgels. As shown in panels (**a,b,c,d**), for the 0.5 L PAH aliquot addition as PAH is titrated into the system in small aliquots, the system goes from a point of high monodispersity before PAH addition (**a**) to a point of minimum radius (**b**); then, as more PAH is added to the system, the radius distribution significantly increases with a shift to extremely high radii indicative of aggregation (**c,d**). As shown in panels (**e,g,h**), for the 5.0 L PAH aliquot addition as PAH is titrated into the system in large aliquots, the system goes from a point of high monodispersity before PAH addition (**e**) to a point of minimum radius upon the first addition of PAH (**f**) and to a point of high radius and high monodispersity (**g,h**), indicative of osmotic/Coulombic swelling. [AAc]/[NH2] for (**b**) is 0.67, (**c**) is 0.19, (**d**) is 0.13, (**f**) is 0.27, (**g**) is 0.067, (**h**) is 0.033.

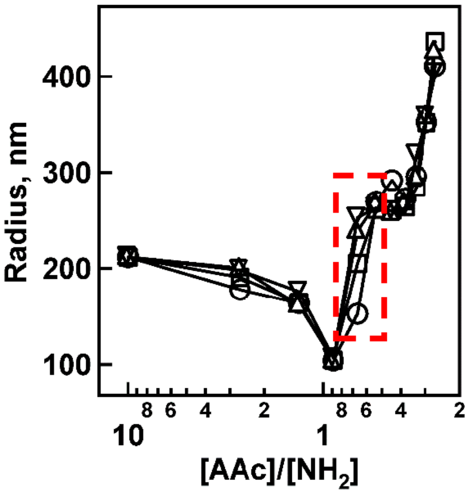

Figure S2. Time-dependent nanogel titration plot for the 2% BIS and 10% AAc nanogels upon the addition of 0.5 μL PAH aliquots at 0 (○), 210 (□), 420 (△), and 630 (▽) s. Note that red-dashed box shows time-dependent kinetics.

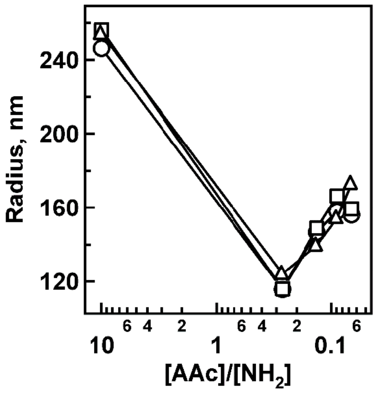

Figure S3. Time-dependent nanogel titration plot for the 2% BIS and 10% AAc nanogels upon the addition of 5.0 L PAH aliquots at 0 (○), 210 (□), and 420 (△) s.

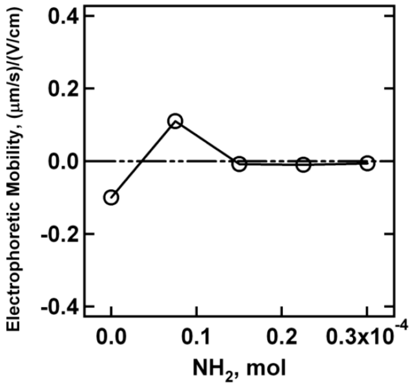

Figure S4. Electrophoretic mobility values as a function of PAH addition for 2% BIS and 98% NIPAm microgels.
